# Late-onset atypical pseudohypoparathyroidism type 1A with intracranial calcification and epilepsy associated with a novel *GNAS* variant: a case report

**DOI:** 10.3389/fendo.2026.1925504

**Published:** 2026-09-17

**Authors:** Daiping Hua, Shang Xiang, Xianfeng Yu, Jingjing Wu, Yequn Wang, Wenming Yang, Han Wang

**Affiliations:** 1 Department of Neurology, The First Affiliated Hospital of Anhui University of Chinese Medicine, Hefei, China; 2 Department of Neurology, Taixing Second People’s Hospital, Taizhou, China; 3 Department of Neurology, The Fifth Affiliated Hospital of Anhui University of Chinese Medicine, Lu’an, China; 4 Key Laboratory of Xin’an Medicine, Ministry of Education, Hefei, China

**Keywords:** cognitive impairment, epilepsy, Fahr syndrome, GNAS gene, intracranial calcification, pseudohypoparathyroidism

## Abstract

Pseudohypoparathyroidism (PHP) type 1A is a rare inherited disorder characterized by resistance to parathyroid hormone and, typically, features of Albright hereditary osteodystrophy. We report a late-onset case in a 59-year-old woman who presented with cognitive decline, behavioral and psychiatric symptoms, gait instability, and seizure-like episodes. Laboratory tests showed hypocalcemia, hyperphosphatemia, and elevated parathyroid hormone levels. Brain imaging revealed symmetrical calcifications in the bilateral basal ganglia, thalami, and periventricular regions, while electroencephalography showed epileptiform discharges. Genetic testing identified a heterozygous missense variant in *GNAS*, c.285C>G: p.Ser95Arg, which has not been previously reported. Based on the clinical manifestations, biochemical abnormalities, neuroimaging findings, and genetic results, the patient was diagnosed with late-onset atypical PHP type 1A. After treatment with calcium supplementation, calcitriol, and levetiracetam, no further seizures occurred, and serum calcium returned to the normal range, although cognitive improvement was limited. This case suggests that calcium-phosphate metabolism and parathyroid hormone levels should be assessed in adults presenting with symmetrical intracranial calcifications, seizures, or cognitive impairment. Genetic testing may further support early diagnosis and help reduce misdiagnosis.

## Introduction

Pseudohypoparathyroidism (PHP) comprises a group of rare inherited disorders characterized by impaired target-organ responsiveness to parathyroid hormone (PTH), typically presenting with hypocalcemia, hyperphosphatemia, and elevated serum PTH levels. The etiology of PHP is heterogeneous. In particular, PHP type 1A is commonly associated with inactivating variants of the *GNAS* gene and may be accompanied not only by disturbances in calcium–phosphate metabolism, but also by features of Albright hereditary osteodystrophy (AHO) and abnormalities involving multiple endocrine axes (1). However, the clinical presentation may be atypical in some patients. This is especially true in adult-onset or late-presenting cases, in which neuropsychiatric manifestations may constitute the initial symptoms, leading to misdiagnosis as epilepsy, cerebrovascular disease, neurodegenerative disorders, or primary Fahr disease (2, 3). Herein, we report the case of a 59-year-old woman who presented predominantly with progressive psychomotor slowing, cognitive decline, and seizures. Neuroimaging revealed symmetrical calcifications involving the bilateral basal ganglia, thalami, and periventricular regions, while laboratory findings were consistent with PTH resistance. Genetic testing identified a heterozygous missense variant in *GNAS*. This case expands the mutational spectrum of *GNAS* and highlights that late-onset atypical PHP type 1A may present chiefly with neurological manifestations, thereby mimicking Fahr syndrome or epileptic disorders. Early recognition of this condition is clinically important for avoiding misdiagnosis, guiding appropriate treatment, and facilitating familial screening.

## Case presentations

The patient was a 59-year-old female farmer who was admitted because of progressive psychomotor slowing for more than 10 months and recurrent episodes of slurred speech accompanied by limb tremors for 5 months. Ten months before admission, her family noticed that she had become increasingly slow to respond, with a vacant facial expression, changes in mood and personality, reduced communication with others, and decreased speech output. She also developed an unsteady gait and walked slowly. She denied hallucinations, loss of consciousness, or limb convulsions. These symptoms progressed gradually. Five months before admission, she began to experience paroxysmal episodes of slurred speech and limb tremors without impaired consciousness. Each episode lasted approximately 10 minutes and was followed by marked weakness of all four limbs. She was evaluated at another hospital, where epilepsy was suspected, and oral levetiracetam was prescribed; however, the underlying etiology remained unclear. After treatment, the severity of the episodes was partially reduced. The patient reported progressive worsening of limb weakness, accompanied by myalgia of all four limbs and poor appetite. During the disease course, she remained conscious but showed psychomotor slowing, gait instability, and slow ambulation. There was no disturbance of consciousness, dysphagia, chills or fever, or urinary or fecal incontinence. Her sleep was fragmented, and her appetite was poor. She had previously slept well. Urination was normal, although bowel control was occasionally impaired.

Her medical history was notable for hypertension, for which she was taking felodipine sustained-release tablets, 5 mg orally once daily. She had a history of cerebral infarction 1 year earlier and had been taking aspirin and atorvastatin calcium regularly. She denied any history of heart disease, infectious diseases such as hepatitis, tuberculosis, or syphilis, surgery, significant trauma, toxin exposure, blood transfusion, or drug or food allergies. Her mother and elder brother had reportedly experienced similar cognitive decline, although both were deceased, and detailed information was unavailable. Her children were healthy.

On physical examination, the patient was 168 cm in height and weighed 62 kg. She showed normal development, with no deformities of the limbs or thoracic cage. The patient was alert, and her speech was articulate; however, her verbal responses were frequently tangential. Assessment of higher cortical functions was impaired, with reduced orientation, calculation ability, and memory. Neurological examination showed that the pupils were equal and round bilaterally, approximately 3 mm in diameter, with brisk light reflexes. The frontal wrinkles and nasolabial folds were symmetrical, and the tongue protruded in the midline. Muscle strength was grade 5 in both upper limbs and grade 4 in both lower limbs. Muscle tone and deep tendon reflexes were normal. Pathological reflexes were absent. Superficial and deep sensations were intact and symmetrical. Bilateral finger-to-nose testing was accurate and steady. No meningeal signs were present. The Mini-Mental State Examination score was 17.

On admission, routine blood tests, liver and renal function tests, coagulation profile, thyroid function, vitamin B12 level, antinuclear antibody panel, vasculitis-related antibodies, and serological tests for syphilis and hepatitis were all within normal limits. Serum electrolyte analysis showed normal sodium and potassium levels, with hypocalcemia and hyperphosphatemia: serum calcium was 1.86 mmol/L (reference range: 2.00–2.65 mmol/L), and phosphorus was 1.73 mmol/L (reference range: 0.85–1.51 mmol/L). PTH level was 86 pg/mL (reference range: 8–40 pg/mL). Lumbar puncture was performed, and cerebrospinal fluid analysis showed normal cell counts and protein levels. Cerebrospinal fluid 14-3–3 protein testing was negative. Ambulatory electroencephalography revealed asymmetric increases in slow-wave activity, with occasional isolated high-amplitude sharp–spike-and-slow-wave complexes over the right frontocentral to anterior temporal regions. Cranial computed tomography (CT) and magnetic resonance imaging (MRI) both revealed symmetric calcifications in the bilateral thalami, basal ganglia, and periventricular regions (**Figure 1**). Given the presentation of epilepsy with cognitive impairment and the exclusion of infectious, inflammatory, and other acquired causes, along with a suggestive family history and symmetric intracranial abnormal calcifications, a hereditary metabolic encephalopathy was considered highly probable. Differential diagnoses included pseudohypoparathyroidism and Fahr disease. Whole-exome sequencing identified a heterozygous missense variant in *GNAS*, NM_080425.4: c.285C>G (p.Ser95Arg), located in exon 1. The corresponding genomic coordinate was chr20: 57428605 based on the hg19 reference genome. The variant was confirmed by Sanger sequencing in the patient (**Figure 2**). Its reported allele frequency in the East Asian population database was 0.000111383. The variant was not listed in ClinVar at the time of reporting. In silico analyses predicted a potentially deleterious effect, with SIFT predicting a damaging effect, PolyPhen-2 predicting a probably damaging effect, and a REVEL score of 0.539. However, the clinical laboratory classified the variant as a variant of uncertain significance, and no specific American College of Medical Genetics and Genomics and the Association for Molecular Pathology (ACMG/AMP) evidence codes were assigned. Taken together with the patient’s clinical manifestations, neuroimaging findings, and laboratory results, a diagnosis of late-onset atypical PHP type 1 was considered most likely. Further evaluations, including serum follicle-stimulating hormone, luteinizing hormone, adrenocorticotropic hormone, growth hormone, estradiol, and blood glucose levels, were all within normal ranges.

**Figure 1:**
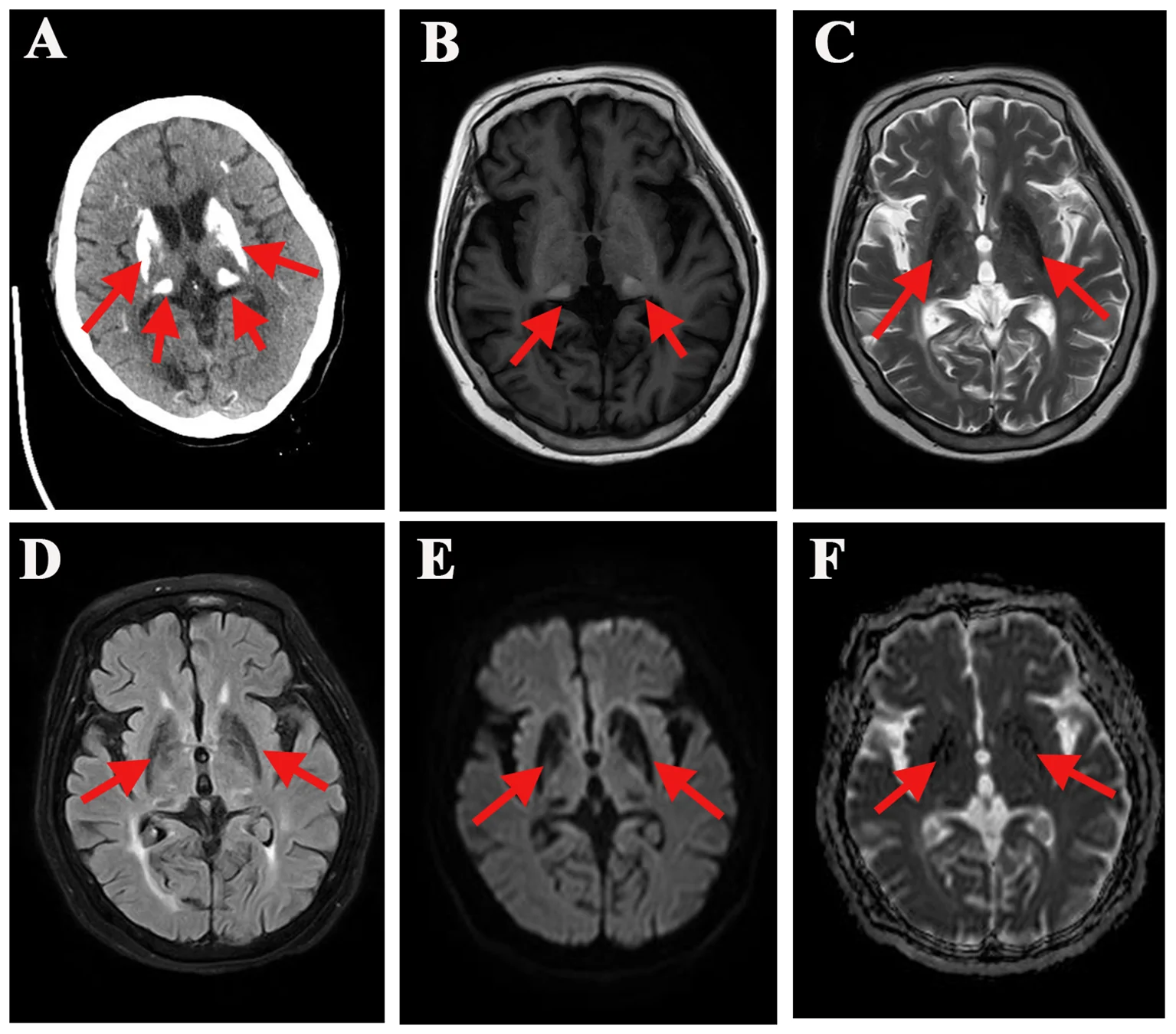
Brain imaging findings. **(A)** Head CT shows symmetrical calcified lesions in the bilateral thalami and basal ganglia regions (red arrows). **(B–F)**: Brain MRI shows symmetrical patchy and strip-like signal abnormalities in the bilateral thalami and basal ganglia regions, mainly presenting as slightly short T1 and slightly short T2 signals. Fluid-attenuated inversion recovery (FLAIR) shows mixed high and low signals; diffusion-weighted imaging (DWI) mainly shows low signals, and apparent diffusion coefficient (ADC) shows mixed signals (red arrows).

**Figure 2:**
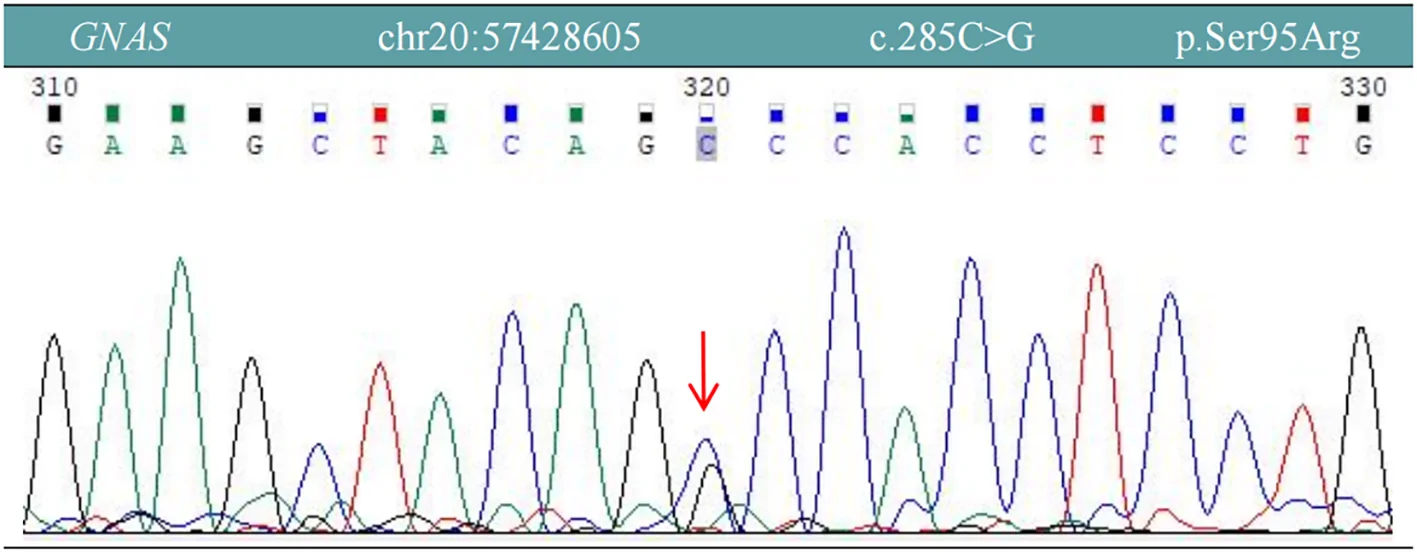
A heterozygous missense variant in the *GNAS* gene, c.285C>G: p.Ser95Arg, located in exon 1.

The patient was commenced on oral calcium carbonate plus vitamin D_3_ (500 mg three times daily) and calcitriol (0.25 μg three times daily) for calcium supplementation, along with levetiracetam (0.5 g twice daily) for seizure control. At the 3-month follow-up, the patient had experienced no further epileptic seizures, and her cognitive level remained unchanged from baseline. Repeat serum electrolyte assessment showed calcium of 2.16 mmol/L (reference range: 2.00–2.65 mmol/L), phosphorus of 1.63 mmol/L (reference range: 0.85–1.51 mmol/L), and PTH of 51 pg/mL (reference range: 8–40 pg/mL). The dosages of calcium carbonate plus vitamin D3 and calcitriol were adjusted to 500 mg twice daily and 0.25 μg twice daily, respectively, while levetiracetam was maintained at the original dose for ongoing antiepileptic therapy. Given the patient’s stable condition, long-term regular medication adherence and periodic monitoring of serum electrolytes and PTH levels were recommended to prevent recurrence of hypocalcemia or the risk of hypercalcemia. Additionally, bone mineral density surveillance was advised to evaluate the long-term efficacy of calcium supplementation, thereby ensuring the patient’s quality of life and neurological stability. A clinical timeline is presented in **Figure 3**.

**Figure 3:**
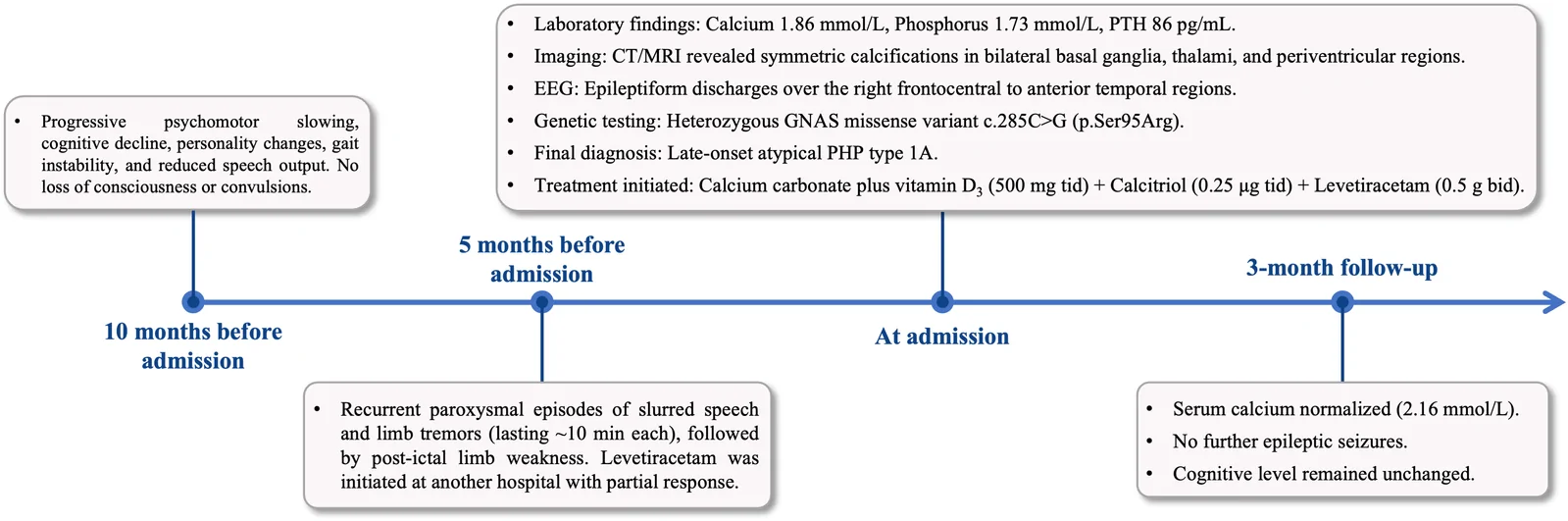
Patient clinical course timeline.

## Discussion

PHP is a group of rare genetic disorders characterized by diminished or absent target organ response to PTH. The global epidemiology remains poorly defined, with a reported prevalence of 1.2 per 100,000 individuals in the Japanese population (4). The classic biochemical profile comprises hypocalcemia, hyperphosphatemia, and compensatory elevation of PTH. Based on clinical phenotypes, patterns of hormone resistance, the presence of AHO features, and underlying molecular genetic mechanisms, PHP can be further classified into several subtypes. Among these, PHP type 1A is most commonly caused by inactivating variants in the maternal allele of the *GNAS* gene. *GNAS* encodes the α-subunit of the stimulatory G protein (*Gsα*), which participates in signal transduction of multiple *Gsα*-coupled receptors and plays a critical role in hormonal pathways involving PTH, thyroid-stimulating hormone, gonadotropins, and growth hormone-releasing hormone. Consequently, dysfunction of *GNAS* not only leads to PTH resistance but may also result in involvement of various endocrine axes and the development of AHO-related features (5).

In the present case, the patient lacked typical AHO features and resistance involving multiple endocrine axes. The disease manifested relatively late, with cognitive decline and seizures as the predominant clinical features. Although this presentation resembles the PHP1B phenotype, which is typically associated with methylation abnormalities at the *GNAS* locus, PHP1A is clinically heterogeneous, and neither a complete AHO phenotype nor resistance to multiple hormones is invariably present. On the basis of the biochemical evidence of PTH resistance and the identification of a heterozygous coding *GNAS* variant, NM_080425.4: c.285C>G (p.Ser95Arg), atypical PHP1A was considered the leading clinical diagnosis. The variant is located in exon 1 and affects the N-terminal coding region of Gsα. Although the substitution could potentially alter Gsα structure or signaling, its functional consequences have not been experimentally established. It was rare in the East Asian population database and was not listed in ClinVar at the time of testing.

The prominent clinical feature in this case was the initial presentation with neurological symptoms. Over the preceding 10 months, the patient had gradually developed cognitive decline, affective and personality changes, reduced speech output, and gait instability, followed by paroxysmal episodes of slurred speech and limb tremors. Ambulatory electroencephalography showed occasional high amplitude sharp spike and slow wave discharges over the right frontocentral to anterior temporal regions, supporting the presence of epileptiform activity. Hypocalcemia can increase neuromuscular excitability and may lead to limb paresthesia, myalgia, spasms, or even epileptic seizures. This effect has been attributed, at least in part, to reduced release of the inhibitory neurotransmitter γ-aminobutyric acid (6). Persistent disturbances of calcium-phosphate metabolism may also promote intracranial calcium deposition, thereby contributing to cognitive impairment, movement abnormalities, and seizures (7). Although the patient had initially received antiseizure medication, seizures continued to occur. After treatment with calcium supplementation and calcitriol, serum calcium normalized, and no further seizures were reported during the subsequent 3-month follow-up. This therapeutic response further supports the important contribution of hypocalcemia and PTH resistance to the patient’s seizure manifestations.

Symmetric intracranial calcification served as a pivotal diagnostic clue in the present case. Cranial imaging revealed bilateral symmetric calcifications involving the basal ganglia, thalami, and periventricular regions. Basal ganglia calcification is a radiographic finding associated with a wide spectrum of disorders, including Fahr disease, hypoparathyroidism, PHP, mitochondrial diseases, infectious conditions, intoxications, cerebrovascular disease, and other hereditary metabolic disorders (8). In patients presenting with bilateral symmetric calcifications of the basal ganglia and thalami accompanied by cognitive decline, epilepsy, neuropsychiatric abnormalities, or movement disorders, Fahr syndrome or Fahr disease is often the initial clinical consideration. However, the diagnosis of Fahr disease typically requires the exclusion of abnormal calcium-phosphorus metabolism and other secondary etiologies (9). In the present case, the presence of frank hypocalcemia, hyperphosphatemia, and elevated PTH levels suggested that the intracranial calcifications were more likely secondary in nature. Genetic analysis identified a heterozygous missense variant in the *GNAS* gene, c.285C>G (p.Ser95Arg), located in exon 1, which has not been previously reported in the literature. In silico prediction tools classified this variant as damaging by SIFT and probably damaging by PolyPhen-2, with a REVEL score of 0.539, suggesting that it may adversely affect protein function. Taken together with the unequivocal evidence of PTH resistance, symmetric intracranial calcifications, epilepsy, and cognitive impairment, as well as the exclusion of infectious, inflammatory, prion-related, and other etiologies, this variant demonstrated strong clinical relevance. Accordingly, a diagnosis of PHP was established.

The present case exhibits several atypical features relative to classic PHP type 1A. Classic PHP type 1A typically presents during childhood or adolescence and is characterized by AHO phenotypes, including short stature, a rounded face, obesity, brachydactyly, subcutaneous ossifications, and intellectual disability. Additionally, it may be accompanied by resistance to thyroid-stimulating hormone, gonadotropins, or growth hormone-releasing hormone (10). In contrast, the patient in this case had normal height and weight with unremarkable development, absence of brachydactyly, chest wall deformities, limb abnormalities, or overt subcutaneous ossifications; and thyroid function, gonadotropic axis, adrenocorticotropic hormone, growth hormone levels, and blood glucose were all within normal limits. Several factors may account for this atypical presentation, including the type and location of the *GNAS* variant, tissue-specific imprinting effects, residual Gsα activity, and the patient’s individual genetic background. Parental testing was not performed; therefore, the parental origin of the variant could not be determined. The variant’s origin accordingly remains unknown. The diagnosis at 59 years of age may reflect relatively mild PTH resistance or a prolonged subclinical course rather than late onset of the underlying molecular abnormality. Partial or tissue-specific PTH resistance may have remained compensated for years through increased PTH secretion. Age-related changes in vitamin D status, renal function, calcium intake, bone turnover, or endocrine reserve may have subsequently impaired this compensation, leading to overt hypocalcemia and hyperphosphatemia. Prolonged calcium-phosphate imbalance may have contributed to progressive calcification of the basal ganglia and thalami. These lesions may remain clinically silent until their cumulative burden, together with hypocalcemia-induced neuronal hyperexcitability, results in seizures, cognitive decline, and neuropsychiatric symptoms. However, because earlier biochemical data and serial neuroimaging were unavailable, the temporal relationship among PTH resistance, intracranial calcification, and neurological manifestations remains uncertain.

The therapeutic goals for PHP are to correct hypocalcemia, control hyperphosphatemia, normalize PTH levels, and prevent hypercalciuria and associated renal complications. The combination of active vitamin D and calcium supplementation represents the mainstay of treatment (11). In the present case, following the initiation of calcium carbonate plus vitamin D3 (500 mg three times daily) and calcitriol (0.25 μg three times daily), serum calcium levels normalized, and seizure activity was successfully controlled. Subsequently, based on follow-up laboratory findings, the dosages were reduced to twice-daily administration in accordance with the principle of individualized dose titration. During the management of PHP, attention should be directed not only toward the restoration of serum calcium levels but also toward the dynamic monitoring of serum phosphorus, PTH, urinary calcium excretion, renal function, and renal ultrasound imaging. Such surveillance is essential to avoid overtreatment, which may predispose patients to hypercalciuria and nephrolithiasis.

### Conclusion

This case report describes a patient with late-onset, atypical pseudohypoparathyroidism type 1A who presented without classic Albright hereditary osteodystrophy features or resistance involving multiple endocrine axes. The predominant manifestations were progressive cognitive decline, neuropsychiatric disturbances, and seizures, accompanied by symmetric intracranial calcifications on neuroimaging. A novel heterozygous *GNAS* missense variant, c.285C>G (p.Ser95Arg), was identified; however, its pathogenicity remains uncertain. Although the variant was rare and predicted to be deleterious by in silico tools, these findings are insufficient to establish causality. In the absence of functional studies, *GNAS* methylation analysis, parental testing, and specific ACMG/AMP evidence codes, the variant should be considered potentially clinically relevant rather than a confirmed molecular cause of the patient’s phenotype. This case highlights the phenotypic heterogeneity of PHP and the diagnostic importance of considering PTH resistance in adults with intracranial calcifications, cognitive impairment, and seizures, even in the absence of typical AHO features. Further family-based studies, functional analyses, and additional cases are needed to clarify the genotype–phenotype relationship.

### Patient perspective

The patient’s daughter stated: “At first, my mother only showed memory decline and slower responses, but later she developed unsteady gait, slurred speech, and limb tremors. Genetic testing eventually confirmed a rare inherited disorder. Although the diagnosis came as a surprise to us, it also provided a clear direction for treatment. We will continue to support her with regular treatment and follow-up.”

## Data Availability

The original contributions presented in the study are included in the article/supplementary material, further inquiries can be directed to the corresponding author/s.
